# Supplementary material for: HOX epimutations driven by maternal SMCHD1/LRIF1 haploinsufficiency trigger homeotic transformations in genetically wildtype offspring
Source: Nat Commun. 2022 Jun 23;13:3583. doi: 10.1038/s41467-022-31185-8 (PMC9226161; doi:10.1038/s41467-022-31185-8)
Supplement: Supplementary file 1 — Supplementary Information [file 41467_2022_31185_MOESM1_ESM.pdf]

HOX epimutations driven by maternal SMCHD1/LRIF1 haploinsufficiency trigger homeotic transformations in genetically wildtype offspring

Shifeng Xue, Thanh Thao Nguyen Ly, Raunak S. Vijayakar, Jingyi Chen, Joel Ng, Ajay S. Mathuru, Frederique Magdinier, Bruno Reversade

Supplementary information

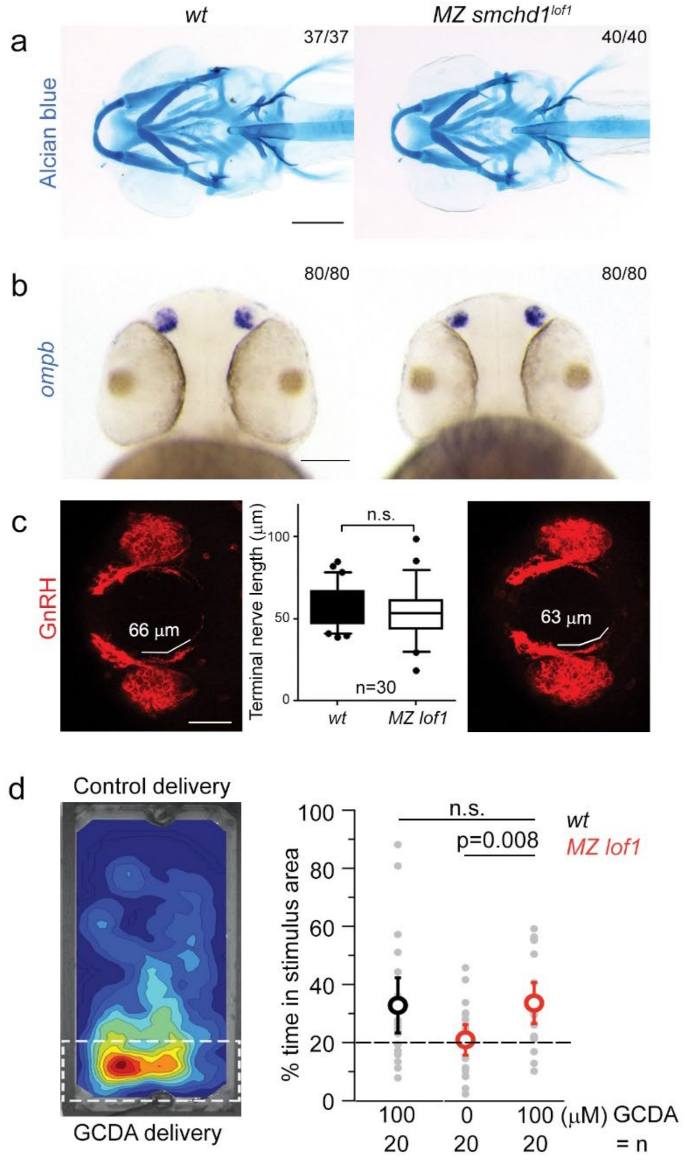

Supplementary Figure 1. ***MZ smchd1*** **null fish do not model Bosma arhinia microphthalmia syndrome**

a, Alcian blue staining shows no difference in craniofacial cartilage development at 5 dpf. **b**, The olfactory epithelium forms normally in *MZ lof1* fish as evidenced by *ompb* expression at 2 dpf, an olfactory epithelium marker. For (**a**) and (**b**), numbers at top right corner represent numbers of embryos with this phenotype (eg. 80 out of 80). Scale bar = 200 µm. **c**, Immunofluorescence of gonadotropic releasing hormone (GnRH) in 33 hpf embryos. Measurements of the terminal nerves marked in white reveal no differences in lengths between *wt* and *MZ lof1* fish. Boxes extend from 25th to 75th percentiles, lines in the centre of boxes represent median, whiskers represent 5th to 95th percentile, n = number of embryos measured per genotype, 2 measurements per embryo (left and right). P values were calculated by 2-tailed unpaired Student's t test. Scale bar = 100 µm. **d**,

Representative trace of a *MZ lof1* larva spending more time in the area near to attractive odor stimulant (glycochenodeoxycholic acid, GCDA) (left). Quantification of olfactory assay shows that *MZ lof1* fish can detect and respond to an odor stimulus (right). Whiskers show 95% confidence intervals. P values were calculated by 2-tailed paired Student's t test.

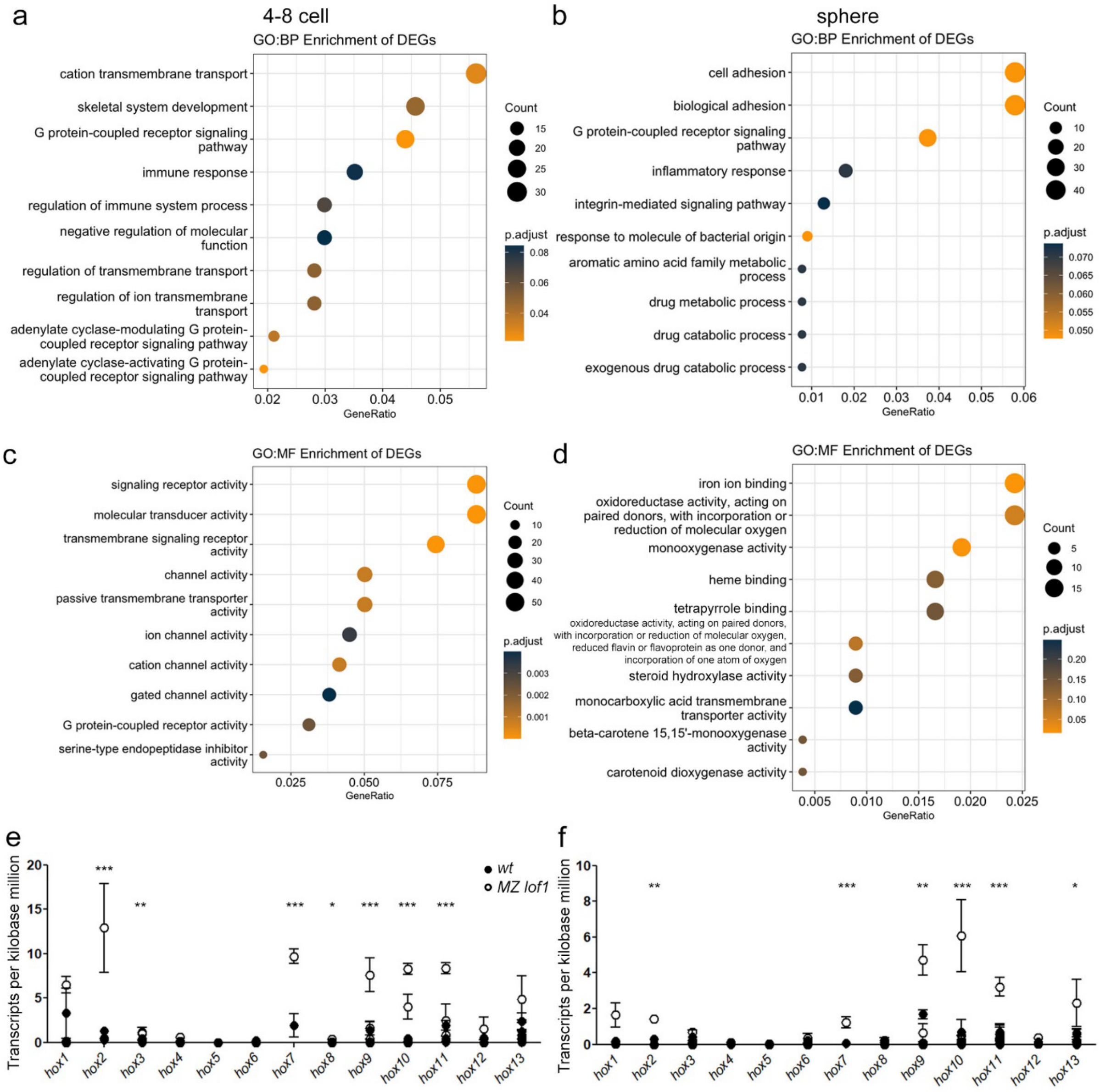

Supplementary Figure 2. Analysis of RNA-sequencing of 4-8-cell and sphere stage embryos.

a-b, Gene ontology (GO) enrichment for biological process (BP) of differentially enriched genes (DEGs) in 4-8-cell (**a**) and sphere stage (**b**) embryos. **c-d**, Gene ontology (GO) enrichment for molecular function (MF) of DEGs in 4-8-cell (**c**) and sphere (**d**) embryos. **e-f**, Transcripts per kilobase million of *hox* genes from RNA-sequencing of 4-8-cell embryos (**e**) or sphere stage embryos (**f**). Data are presented as mean values +/- SD. n = 3 biological samples. * represent FDR-adjusted p values from DESeq2. *p<0.05, **p<0.01, ***p<0.001

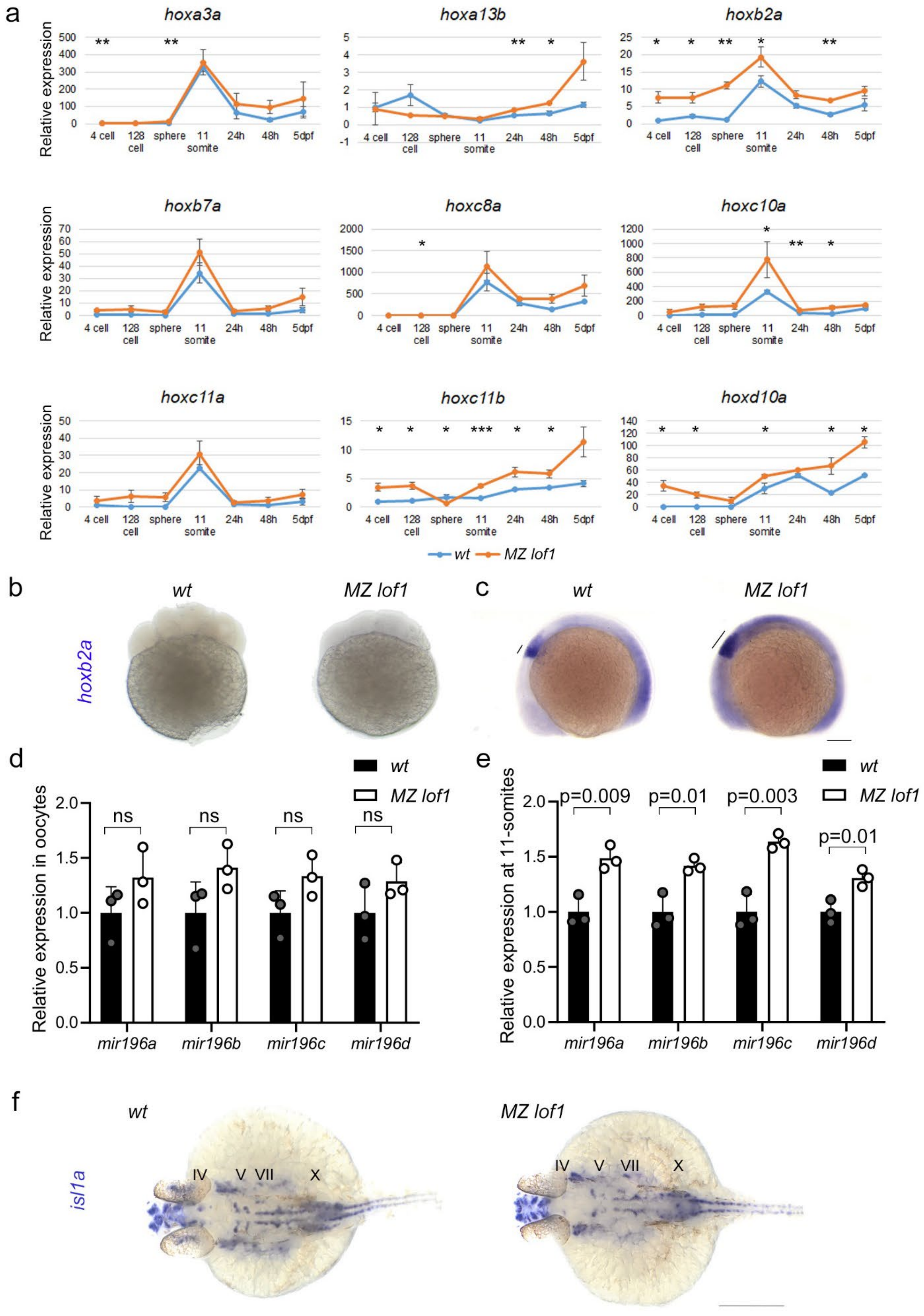

a
hoxa3a
hoxa13b
hoxb2a
hoxb7a
hoxc8a
hoxc10a
hoxc11a
hoxc11b
hoxd10a
Relative expression
4 cell
128 cell
sphere
11 somite
24h
48h
5dpf
wt
MZ lof1
b
c
hoxb2a
d
Relative expression in oocytes
ns
mir196a
mir196b
mir196c
mir196d
e
Relative expression at 11-somites
p=0.009
p=0.01
p=0.003
p=0.01
f
isl1a
IV
V
VII
X

Supplementary Figure 3. Analysis of *hox* expression changes over time

a, qPCR analysis of hox expression in *wt* and *MZ lof1* embryos over time. Expression of *hox* gene in *wt* at 4-cell stage is set at 1. Data represents mean ± standard deviation. **b-c**, In situ hybridisation (ISH) of *hoxb2a* at 8-cell (**b**) and 11-somite stage (**c**). Expression of *hoxb2a* at 8-cell was not reliably detectable by in situ hybridisation. Scale bar = 200 µm. **d-e**, qPCR of *mir-196* family mature miRNAs in oocytes (**d**) and 11-somite stage (**e**). P values were calculated by 2-tailed unpaired Student's t test. Data represents mean ± standard deviation of 3 biological replicates. *p<0.05, **p<0.01, ***p<0.001. **f**, ISH of *isl1a* of cranial nerves in the hindbrain do not show a patterning difference between *wt* and *MZ lof1* embryos at 30 hpf. n = 40 *wt*, 80 *MZ lof1* embryos. Scale bar = 200 µm.

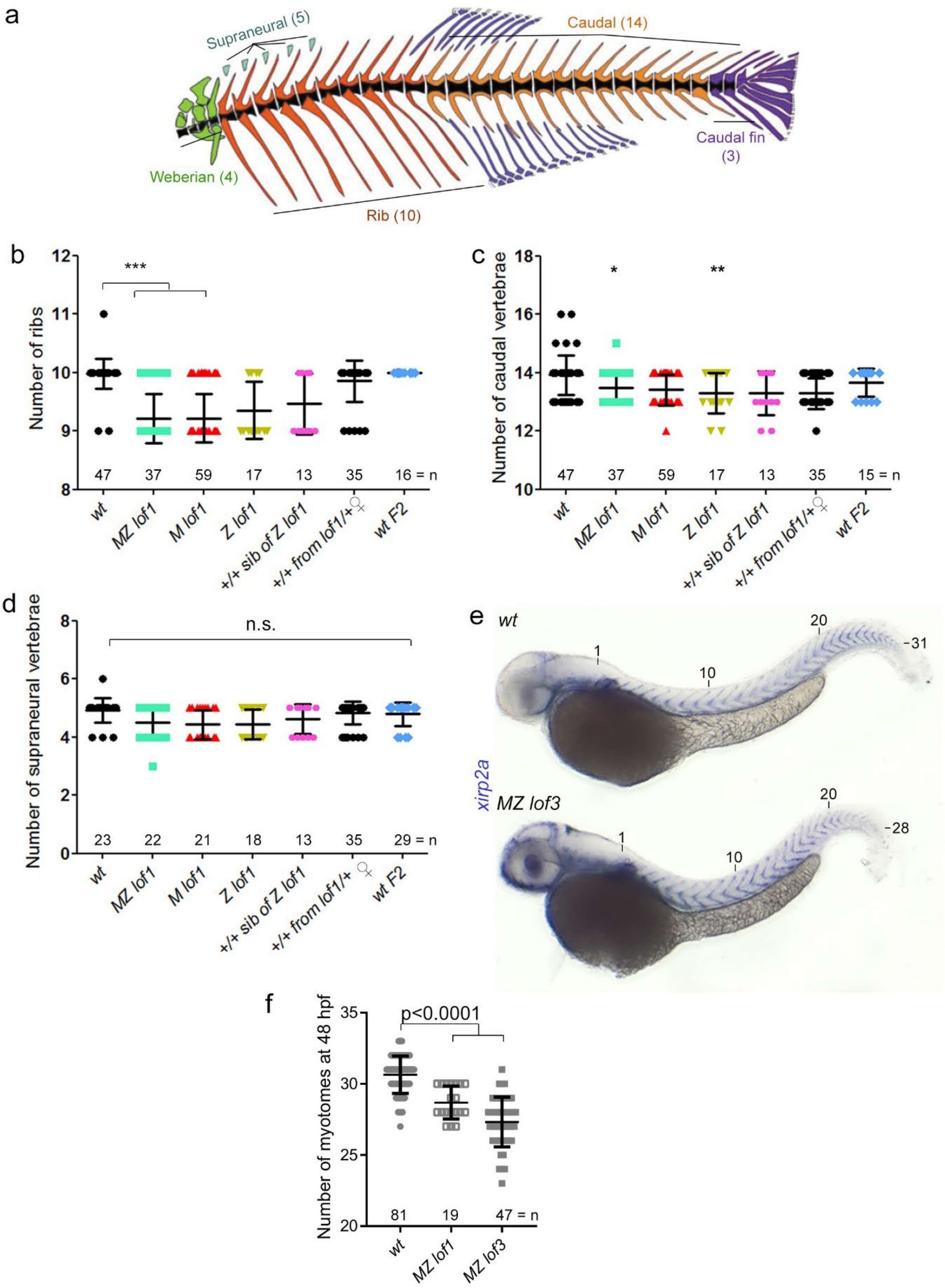
a
Supraneural (5)
Caudal (14)
Weberian (4)
Rib (10)
Caudal fin (3)
b
Number of ribs
***
47 37 59 17 13 35 16 = n
wt
MZ lof1
M lof1
Z lof1
+/+ sib of Z lof1
+/+ from lof1/+ ♀
wt F2
c
Number of caudal vertebrae
*
**
47 37 59 17 13 35 15 = n
d
Number of supraneural vertebrae
n.s.
23 22 21 18 13 35 29 = n
e
wt
xirp2a
MZ lof3
f
Number of myotomes at 48 hpf
p<0.0001
81 19 47 = n
wt
MZ lof1
MZ lof3

Supplementary Figure 4. Breakdown of vertebral number changes

a, Schematic of zebrafish skeleton adapted from Bird and Mabee (2003)[41]. Copyright © 2003 Wiley-Liss, Inc. **b-d**, Number of ribs (**b**), caudal vertebrae (**c**) and supraneural vertebrae (**d**) in zebrafish from different genetic crosses. Data are presented as mean values +/- SD. P values were calculated by the Kruskal-Wallis test followed by Dunn's Multiple Comparison Test. **e**, Reduction in number of myotomes as shown by somitic myotome boundary marker *xirp2a* at 48 hpf. Scale bar = 500 µm. **f**, Quantification shows a reduction in the number of myotomes in the *MZ lof* larvae. Data are presented as mean values +/- SD. P values were calculated by the Kruskal-Wallis test followed by Dunn's Multiple Comparison Test. *p<0.05, **p<0.01, ***p<0.001.

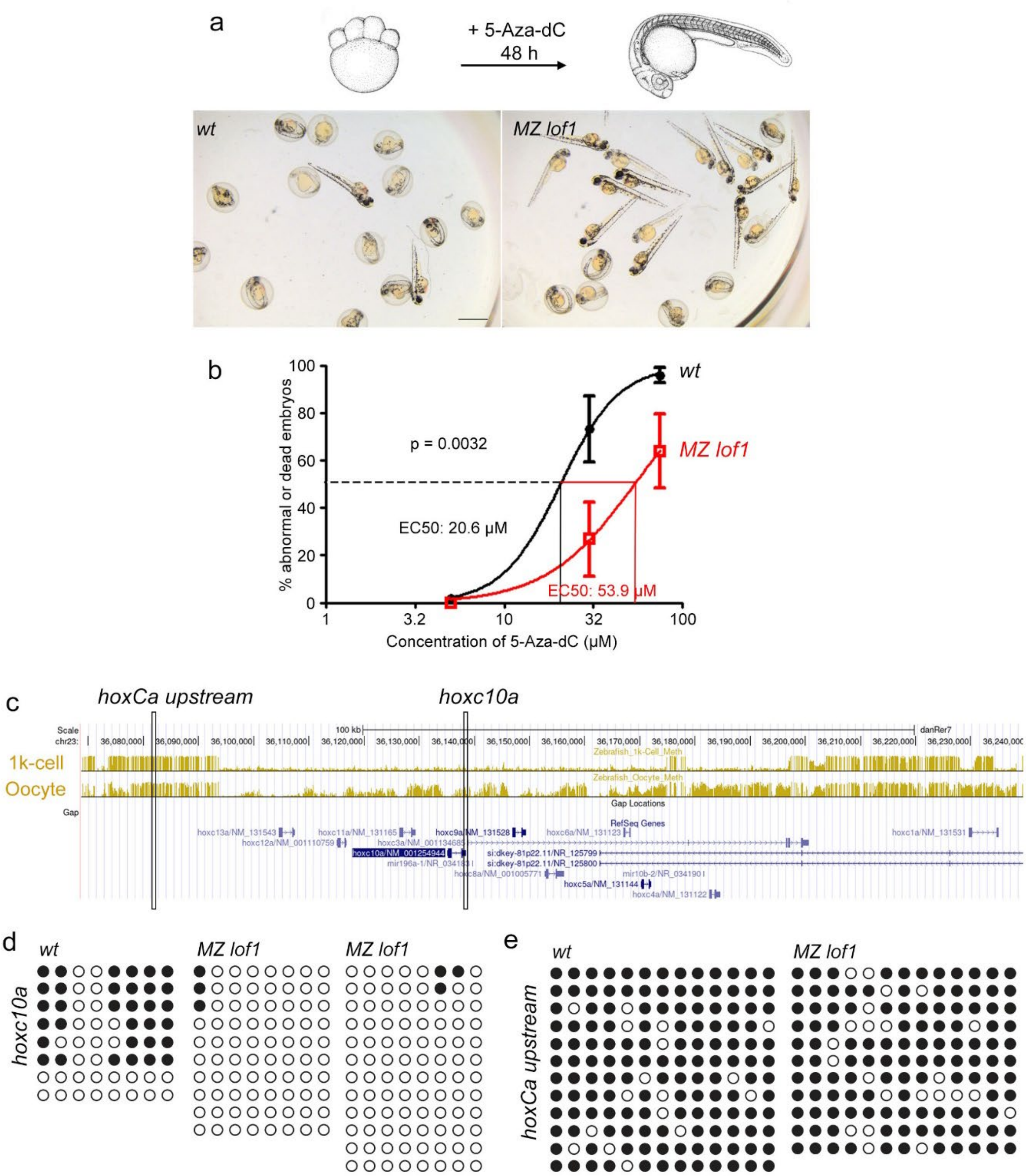

Supplementary Figure 5. DNA methylation analysis in zebrafish embryos

a, *wt* or *MZ smchd1 lof1* embryos were treated with DNA methylation inhibitor 5-Aza-dC for 2 days. Representative images of embryos treated with 30 μM 5-Aza-dC are shown. Defects observed include edema, developmental delay and axis truncation. Scale bar = 1 mm. **b**, *MZ lof1* embryos show enhanced survival and fewer developmental defects than *wt*. n = 121 *wt*, 123 *MZ smchd1 lof1* embryos. Data are presented as mean values +/- SEM. Curves were fitted with least squares regression. P values were calculated by extra sum-of-squares F test. **c**, Location of DNA methylation analysis in the *hoxCa*

locus. DNA methylation data of wildtype embryos from Jiang et al. (2013) [19]. **d**, Replicates of DNA methylation analysis at *hoxc10a*. **e**, DNA methylation analysis at *hoxCa* upstream. Hypomethylation in mutant embryos was only observed at positions in *hox* loci which undergo dynamic changes during embryogenesis.

References

41. Bird, N. C. & Mabee, P. M. Developmental morphology of the axial skeleton of the zebrafish, Danio rerio (Ostariophysi: Cyprinidae). *Dev. Dyn.* **228**, 337–357 (2003).
