# Supplementary material for: HOX epimutations driven by maternal SMCHD1/LRIF1 haploinsufficiency trigger homeotic transformations in genetically wildtype offspring
Source: Nat Commun. 2022 Jun 23;13:3583. doi: 10.1038/s41467-022-31185-8 (PMC9226161; doi:10.1038/s41467-022-31185-8)
Supplement: Supplementary file 3 — Description of Additional Supplementary Files [file 41467_2022_31185_MOESM3_ESM.docx]

**HOX epimutations driven by maternal SMCHD1/LRIF1 haploinsufficiency trigger homeotic transformations in genetically wildtype offspring**

Shifeng Xue, Thanh Thao Nguyen Ly, Raunak S. Vijayakar, Jingyi Chen, Joel Ng, Ajay S. Mathuru, Frederique Magdinier, Bruno Reversade

**Description of additional supplementary files**

**Supplementary Movie 1. *smchd1^-/-^* respond to *Schreckstoff* (olfactory alarm cue)**Example of a homozygous *MZ lof1* fish responding to the alarm substance like wild type fish as described previously^42^. *Schreckstoff* was introduced via an automated delivery valve as indicated by the light at the top left-hand corner. Fish respond to this alarm substance by darting around and eventually freezing.

**Supplementary Data 1. List of primers used in study**

**Supplementary Data 2. Differentially expressed genes in both 4-8-cell and sphere stages.**

**References**

1. Mathuru, A. S. et al. Chondroitin fragments are odorants that trigger fear behavior in fish. *Curr. Biol.* **22**, 538–544 (2012).
