# Supplementary material for: HOX epimutations driven by maternal SMCHD1/LRIF1 haploinsufficiency trigger homeotic transformations in genetically wildtype offspring
Source: Nat Commun. 2022 Jun 23;13:3583. doi: 10.1038/s41467-022-31185-8 (PMC9226161; doi:10.1038/s41467-022-31185-8)
Supplement: Supplementary file 7 — Reporting Summary [file 41467_2022_31185_MOESM7_ESM.pdf]

nature portfolio

Corresponding author(s): Shifeng Xue, Bruno Reversade

Last updated by author(s): May 10, 2022

Reporting Summary

Nature Portfolio wishes to improve the reproducibility of the work that we publish. This form provides structure for consistency and transparency in reporting. For further information on Nature Portfolio policies, see our Editorial Policies and the Editorial Policy Checklist.

Statistics

For all statistical analyses, confirm that the following items are present in the figure legend, table legend, main text, or Methods section.

n/a	Confirmed	
☐	☒	The exact sample size (*n*) for each experimental group/condition, given as a discrete number and unit of measurement
☐	☒	A statement on whether measurements were taken from distinct samples or whether the same sample was measured repeatedly
☐	☒	The statistical test(s) used AND whether they are one- or two-sided<br>*Only common tests should be described solely by name; describe more complex techniques in the Methods section.*
☒	☐	A description of all covariates tested
☐	☒	A description of any assumptions or corrections, such as tests of normality and adjustment for multiple comparisons
☐	☒	A full description of the statistical parameters including central tendency (e.g. means) or other basic estimates (e.g. regression coefficient) AND variation (e.g. standard deviation) or associated estimates of uncertainty (e.g. confidence intervals)
☐	☒	For null hypothesis testing, the test statistic (e.g. *F*, *t*, *r*) with confidence intervals, effect sizes, degrees of freedom and *P* value noted<br>*Give P values as exact values whenever suitable.*
☒	☐	For Bayesian analysis, information on the choice of priors and Markov chain Monte Carlo settings
☒	☐	For hierarchical and complex designs, identification of the appropriate level for tests and full reporting of outcomes
☒	☐	Estimates of effect sizes (e.g. Cohen's *d*, Pearson's *r*), indicating how they were calculated

Our web collection on statistics for biologists contains articles on many of the points above.

Software and code

Policy information about availability of computer code

Data collection	No software used.
Data analysis	Fish videos were analysed using the "track objects" algorithm in MetaMorph. 6.3. qPCR data was analysed with QuantStudio v1.5.2. Statistics and graphs were generated using Graphpad Prism 8.

For manuscripts utilizing custom algorithms or software that are central to the research but not yet described in published literature, software must be made available to editors and reviewers. We strongly encourage code deposition in a community repository (e.g. GitHub). See the Nature Portfolio guidelines for submitting code & software for further information.

Data

Policy information about availability of data

All manuscripts must include a data availability statement. This statement should provide the following information, where applicable:

- Accession codes, unique identifiers, or web links for publicly available datasets
- A description of any restrictions on data availability
- For clinical datasets or third party data, please ensure that the statement adheres to our policy

The RNA-seq data produced in this study were submitted to GEO under the following accession number: GSE173462 (token: qzwtowmgnvefven), GSE174604 (token: gnklgckshtubhox).

March 2021

Field-specific reporting

Please select the one below that is the best fit for your research. If you are not sure, read the appropriate sections before making your selection.

☒ Life sciences ☐ Behavioural & social sciences ☐ Ecological, evolutionary & environmental sciences

For a reference copy of the document with all sections, see nature.com/documents/nr-reporting-summary-flat.pdf

Life sciences study design

All studies must disclose on these points even when the disclosure is negative.

Sample size	No sample size calculations were performed. Sample sizes were used according to other zebrafish studies.
Data exclusions	No data exclusion.
Replication	All experiments were repeated at least 3 times. Fish were collected from multiple clutches. Data shown include replicates from all clutches. Mice experiments include animals from crosses of different individuals to ensure reproducibility. All attempts at replications were successful.
Randomization	No randomization. Animals were allocated into experimental groups based on their genotype.
Blinding	No blinding was performed. Animals were genotyped prior to analysis. Data reported are not subjective but based on experimental observations.

Reporting for specific materials, systems and methods

We require information from authors about some types of materials, experimental systems and methods used in many studies. Here, indicate whether each material, system or method listed is relevant to your study. If you are not sure if a list item applies to your research, read the appropriate section before selecting a response.

Materials & experimental systems

n/a	Involved in the study	
☐	☒	Antibodies
☐	☒	Eukaryotic cell lines
☒	☐	Palaeontology and archaeology
☐	☒	Animals and other organisms
☐	☒	Human research participants
☒	☐	Clinical data
☒	☐	Dual use research of concern

Methods

n/a	Involved in the study	
☒	☐	ChIP-seq
☒	☐	Flow cytometry
☒	☐	MRI-based neuroimaging

Antibodies

Antibodies used	SMCHD1 (Atlas HPA039441, 1:500), Histone H3 (abcam ab1791, 1:1000), GAPDH (Santa Cruz SC47724, 1:1000), GnRH (Sigma G8294, 1:500)
Validation	SMCHD1: verified with KO zebrafish Western blot (WB) in this manuscript. Histone H3: validated for WB by manufacturer and used in >3000 publications, predicted to work with zebrafish. GAPDH: validated for WB by manufacturer for human protein, used in >2000 publications. GnRH: used for immunofluorescence in Shaw et al. (2017), data which we attempted to validate here.

Eukaryotic cell lines

Policy information about cell lines

Cell line source(s)	Primary fibroblast were derived from patient skin biopsy.
Authentication	Genotype of cell line was verified by Sanger sequencing
Mycoplasma contamination	Cell lines tested negative for mycoplasma
Commonly misidentified lines (See ICLAC register)	None

March 2021

Animals and other organisms

Policy information about studies involving animals; ARRIVE guidelines recommended for reporting animal research

Laboratory animals	AB Danio rerio of both male and female. Adult fish used were between 3 and 18 months old. All mice used were on the C57BL/6 background. Both males and females were used. Adult mice used for generating embryos and pups were between 1.5 and 6 months old. Mice were maintained at 20°C - 25°C, 45–65% humidity, and a light/dark cycle of 14h/10h.
Wild animals	None
Field-collected samples	None
Ethics oversight	Zebrafish were maintained and used according to the Institutional Animal Care and Use Committee of Biological Resource Centre, A*STAR, Singapore (IACUC #161172) and the National University of Singapore (IACUC #BR19-1184). All mice procedures were approved by the Institutional Animal Care and Use Committee of Biological Resource Centre, A*STAR, Singapore (IACUC #201555).

Note that full information on the approval of the study protocol must also be provided in the manuscript.

Human research participants

Policy information about studies involving human research participants

Population characteristics	For FSHD, individuals were clinically assessed by neurologists with expertise in neuromuscular diseases who defined the presence or total absence of clinical signs and evaluated the involvement of the typical groups of muscle usually affected in the disease (facial, shoulder and pelvic girdle, upper and lower limbs and abdominal muscles). All patients were diagnosed at the Department of Medical Genetics, La Timone Hospital Marseille either by Southern blotting or Molecular Combing.
Recruitment	Patients were recruited according to their phenotype and genetic mutations. Controls are randomly selected individuals or patient's relatives. Controls are neither carrier of any genetic mutation in FSHD nor affected by any muscular pathology. Controls were selected in the same age range and sex representation as patients. There was no self selection bias.
Ethics oversight	Written informed consent of individuals were received prior to collection of samples and subsequent analysis for research purposes according to the ethical approvals of the local Institutional Review Boards in Singapore (A*STAR IRB 2019-087, NUS IRB N-20-054E) and France (La Timone Children's Hospital).

Note that full information on the approval of the study protocol must also be provided in the manuscript.
